# Follicular lymphoid hyperplasia of the posterior maxillary site presenting as uncommon entity: a case report and review of the literature

**DOI:** 10.1186/s12903-019-0936-9

**Published:** 2019-11-11

**Authors:** Masato Watanabe, Ai Enomoto, Yuya Yoneyama, Michihide Kohno, On Hasegawa, Yoko Kawase-Koga, Takafumi Satomi, Daichi Chikazu

**Affiliations:** Department of Oral and Maxillofacial Surgery, Tokyo Medical University Hospital, 6-7-1 Nishishinjuku, Shinjuku, Tokyo, 160-0023 Japan

**Keywords:** Follicular lymphoid hyperplasia, Immunohistochemistry, Maxilla, Differential diagnosis, Lymphoma

## Abstract

**Background:**

Follicular lymphoid hyperplasia (FLH) is characterized by an increased number and size of lymphoid follicles. In some cases, the etiology of FLH is unclear. FLH in the oral and maxillofacial region is an uncommon benign entity which may resemble malignant lymphoma clinically and histologically.

**Case presentation:**

We report the case of a 51-year-old woman who presented with an asymptomatic firm mass in the left posterior maxillary site. Computed tomography scan of her head and neck showed a clear circumscribed solid mass measuring 28 × 23 mm in size. There was no evidence of bone involvement. Incisional biopsy demonstrated benign lymphoid tissue. The patient underwent complete surgical resection. Histologically, the resected specimen showed scattered lymphoid follicles with germinal centers and predominant small lymphocytes in the interfollicular areas. Immunohistochemically, the lymphoid follicles were positive for CD20, CD79a, CD10, CD21, and Bcl6. The germinal centers were negative for Bcl2. Based on these findings, a diagnosis of benign FLH was made. There was no recurrence at 1 year postoperatively.

**Conclusions:**

We diagnosed an extremely rare case of FLH arising from an unusual site and whose onset of entity is unknown. Careful clinical and histopathological evaluations are essential in making a differential diagnosis from a neoplastic lymphoid proliferation with a nodular growth pattern.

## Background

Follicular lymphoid hyperplasia (FLH) is characterized as a non-neoplastic lymphoproliferative disease, and is also known as nodular lymphoid lesion and pseudolymphoma. This rare disease is known to affect various organs including the skin, lungs, stomach, breasts, intestine, spleen, pancreas, and liver [1, 2]. In the head and neck region, involvements of the orbit, thyroid, and oral cavity have been demonstrated. Morphologically, the lesion may show the features of FLH. The clinicopathological features of lymphoid hyperplasia in the oral mucosa were initially reported by Adkins in 1973 [3]. The author indicated the hard palate as the most commonly affected site. FLH of the hard palate may be confused clinically and histologically with malignant lymphoma. The condition most commonly affects elderly patients and presents as a painless, slowly growing non-ulcerated mass in the posterior hard palate [1]. The pathogenesis of FLH in the oral cavity is presently unknown. However, it has been suggested that the reactive proliferation is associated with some unknown antigenic stimulation. On the other hand, it may be considered that its occurrence in the submucosal tissue in the oral and maxillofacial areas except in the palate is extremely rare. Although it is also important to have a differential diagnosis from malignant lymphoma or the systemic disease of multicentric lymphadenopathy in this area, a definitive diagnosis of FLH could only be made clinically and histopathologically. We describe the case of a patient with FLH found in the posterior maxilla arising from an uncommon site, and review its histopathological features by immunohistochemical analysis.

## Case presentation

**Fig. 1:**
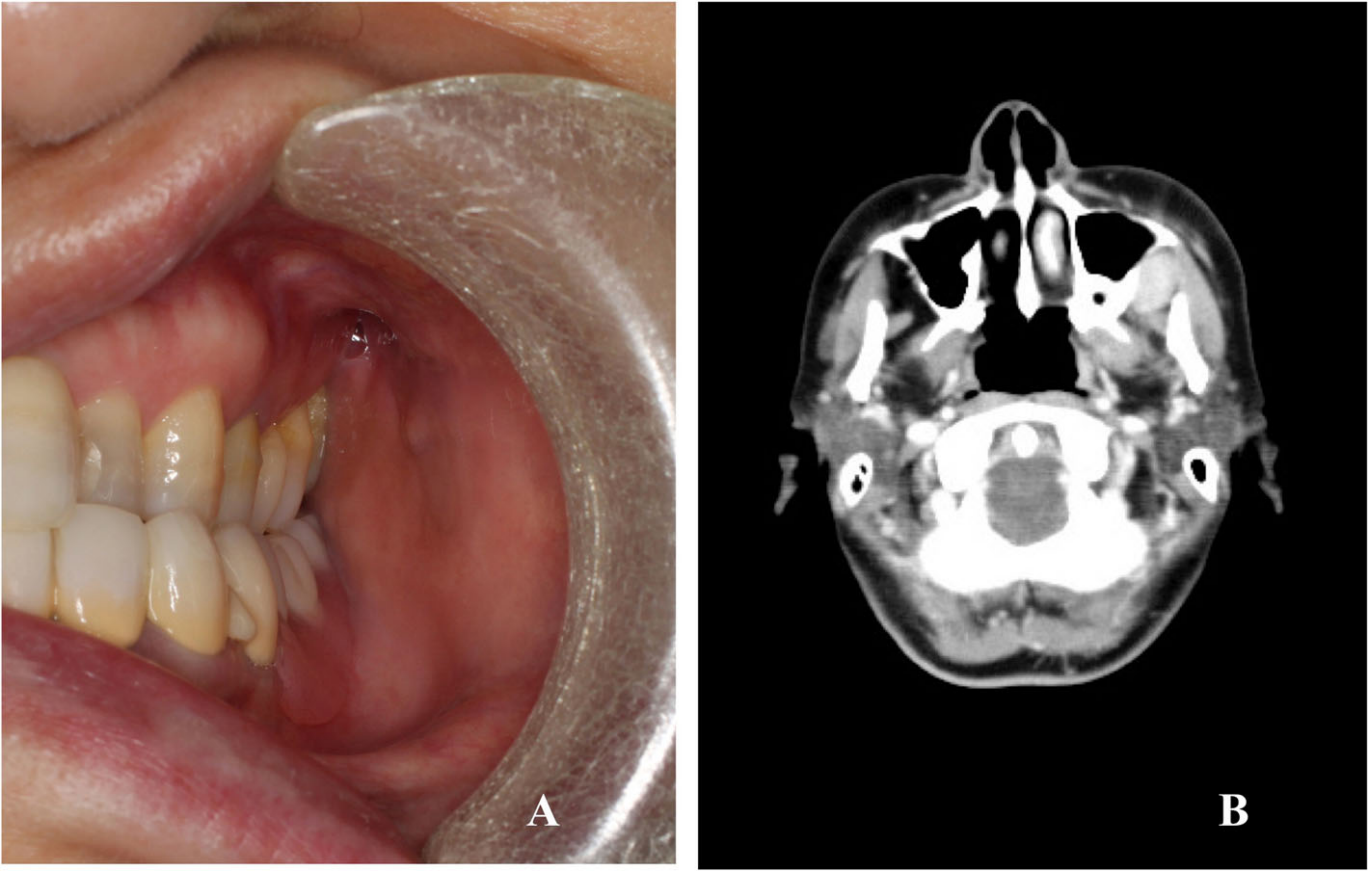
**a** Intraoral appearance of the mass lesion overlying by normal mucosa in the maxillary posterior site. **b** Computed tomography scan showing a clear circumscribed solid mass between the lateral aspect of the posterior maxilla and the anterior region of the masseter muscle

**Fig. 2:**
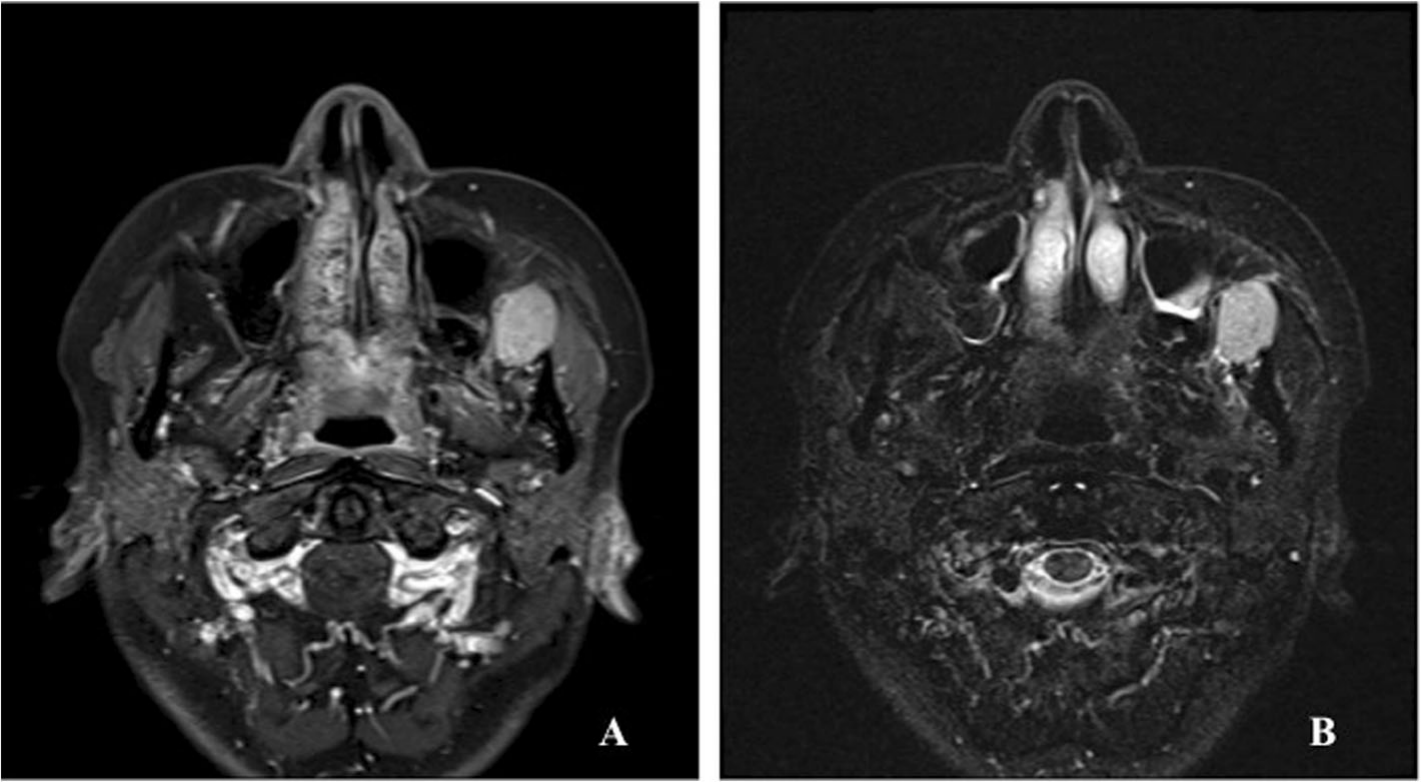
Magnetic resonance imaging scan showing (**a**) homogeneously enhanced signal intensity on a T1-weighted image after gadolinium administration and (**b**) high signal intensity on a Short T1 Inversion Recovery image

A 51-year-old woman was referred to the Department of Oral and Maxillofacial Surgery at Tokyo Medical University Hospital for a closer evaluation and treatment of an asymptomatic firm mass in the left posterior maxillary site. Intraoral examination revealed a palpable mass located in the posterior lateral region of the left maxilla (Fig. 1a). The overlying mucosa was intact and normal in color. The firm mass was not movable. Laboratory data were within the normal limits. Computed tomography (CT) scan of her head and neck showed a clear circumscribed solid mass measuring 28 × 23 mm in size between the lateral aspect of the posterior maxilla and the anterior region of the masseter muscle with no evidence of maxillary bone involvement (Fig. 1b). There was no lymphadenopathy in the cervical region. Magnetic resonance imaging scans revealed homogeneously enhanced signal intensity on T1-weighted images after gadolinium administration (Fig. 2a) and high signal intensity on Short T1 Inversion Recovery images (Fig. 2b). Incisional biopsy demonstrated benign lymphoid tissue. CT examination was conducted in the chest and abdominal region taking into consideration Castleman’s disease as a differential diagnosis. However, systemic lymphadenopathy was not recognized. The submucosal mass was completely excised under general anesthesia. In the course of the surgical resection, the mass was observed to be adjacent to the buccal fat pad.

**Fig. 3:**
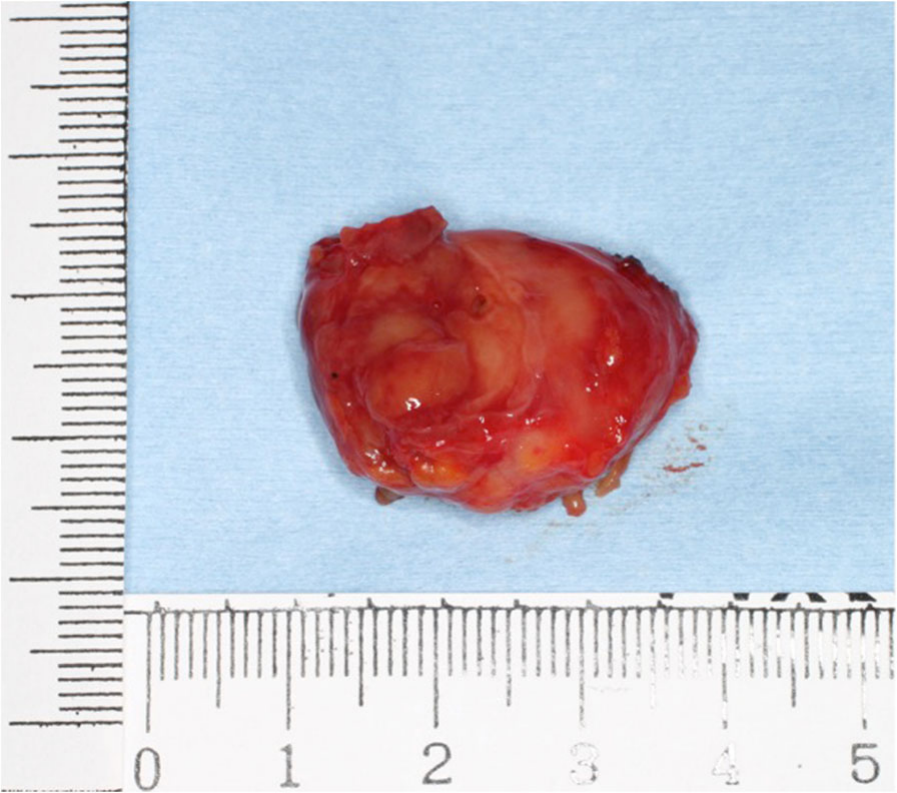
Surgical specimen of the solid mass measuring 38 × 23 × 17 mm in size, which was encapsulated with fibrous tissue

**Fig. 4:**
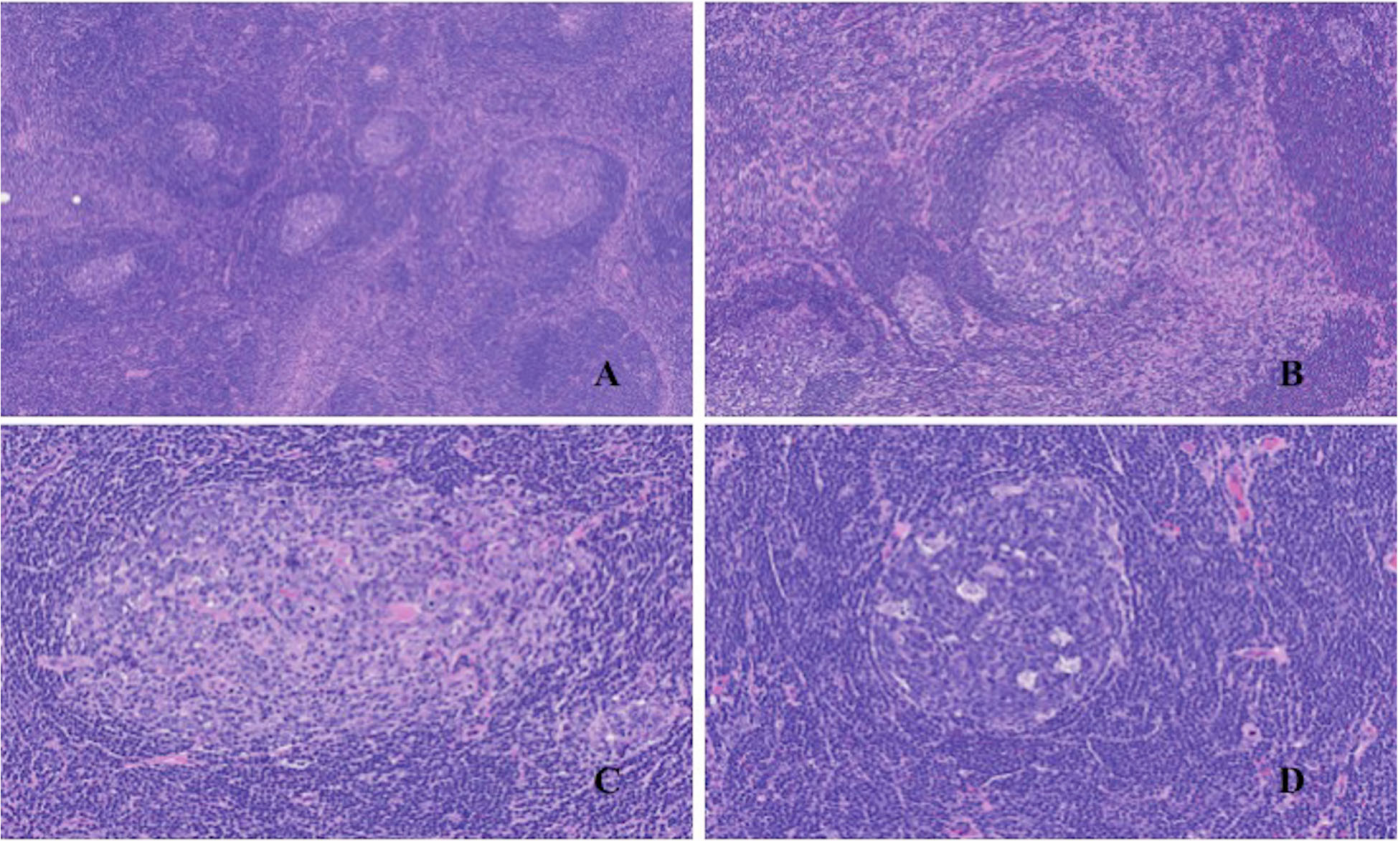
Microscopic appearance of the hyperplastic lymphoid tissue. **a** Scattered lymphoid follicles with germinal centers (hematoxylin and eosin, 5x). **b** A germinal center surrounded by a well-defined mantle zone consisting of small lymphocytes (hematoxylin and eosin, 10x). **c** Follicle center containing a mixture of centrocytes and centroblasts (hematoxylin and eosin, 20x). **d** Tingible body macrophages in the germinal center (hematoxylin and eosin, 20x)

**Fig. 5:**
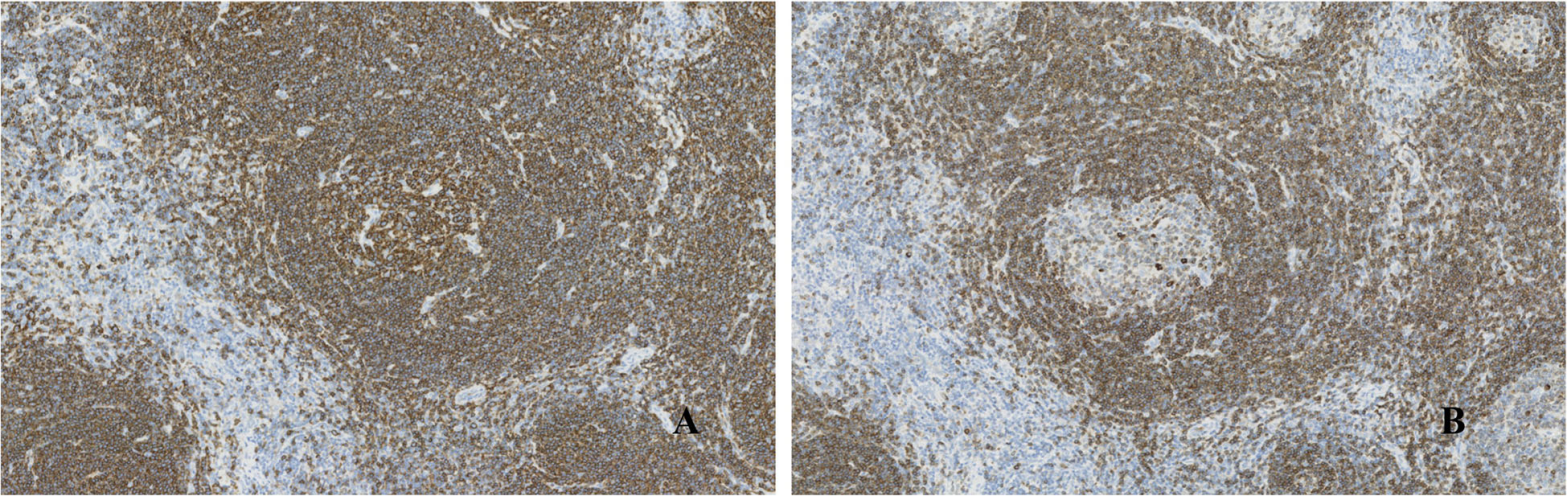
Immunohistochemical features of the lymphoid follicles. **a** The whole areas of lymphoid follicles were positive for CD20 (10x). **b** The lymphoid follicles except the germinal center were positive for CD79a (10x)

**Fig. 6:**
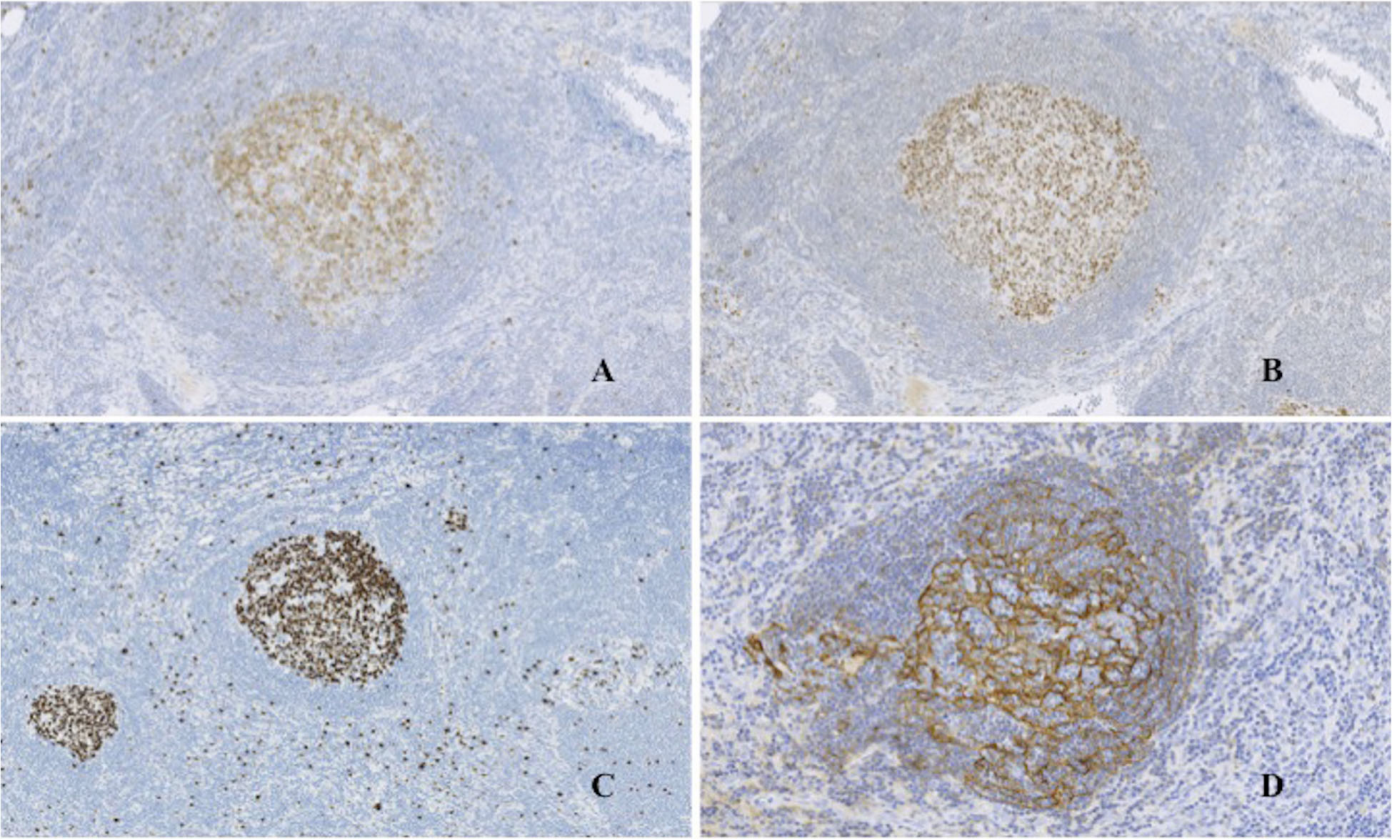
The germinal centers showed positive immunostaining for CD10 (**a**), Bcl6 (**b**), and Ki67 (**c**) (10x). The immunostaining for CD21 revealed a reticular pattern highlighting the follicular dendritic cell networks (**d**) (20x)

**Fig. 7:**
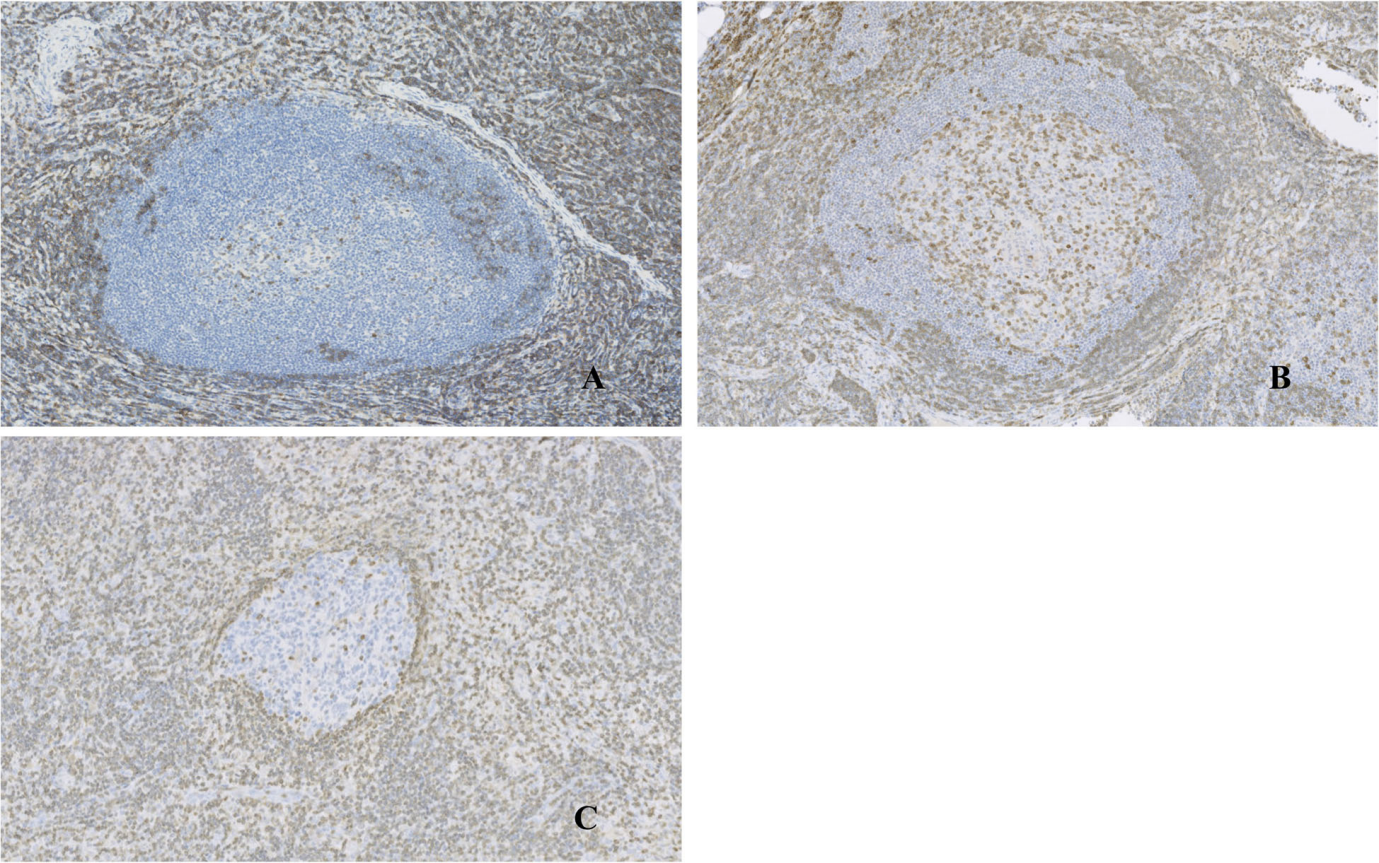
The parafollicular areas showed immunostaining for CD3 (**a**) and CD5 (**b**), and weakly positive immunostaining for Bcl2 (**c**) (10x)

Gross examination showed a solid mass measuring 38 × 23 × 17 mm in size, which was encapsulated with fibrous tissue **(**Fig. 3**)**. Microscopic examination revealed lymphocytic tissue composed of scattered lymphoid follicles with germinal centers and predominant small well-differentiated lymphocytes in the parafollicular area **(**Fig. 4a**)**. The follicles showed various sizes of the germinal centers surrounded by well-defined mantle zones consisting of small lymphocytes **(**Fig. 4b**)**. The germinal center contained a mixture of centrocytes and centroblasts with occasional tingible body macrophages (Fig. 4c and d). The nuclei of the centrocytes exhibited the cleaved type. The centroblasts had relatively large nuclei with prominent nucleoli. Penetration of vessels similarly to the hyaline vascular type in Castleman’s disease was observed in one part of the germinal centers. Immunohistological analysis demonstrated the positivity of lymphoid follicles for CD20 and CD79a **(**Fig. 5**)**. In particular, there were expressions for CD10, Bcl6, and Ki67 only in the germinal centers, whereas there was no expression for Bcl2 **(**Fig. 6a, b, and c**)**. The immunostaining for CD21 was characteristic of a reticular pattern highlighting the follicular dendritic cell networks **(**Fig. 6d**)**. The parafollicular areas expressed CD3 and CD5, and were weakly positive for Bcl2 **(**Fig. 7**)**. Both areas showed CD45RO staining, but conversely not CD15 expression. CD45RO and CD15 images were not shown. Based on these findings, a final diagnosis of FLH was made. The patient has been free of local recurrence 1 year postexcision.

## Discussion and conclusions

FLH is recognized as a non-neoplastic proliferative lymphoid lesion and shows very uncommon features. It is also referred to as pseudolymphoma and nodular lymphoid tissue. FLH involves various organs such as the skin, orbit, nasopharynx, larynx, thyroid, lungs, gastrointestinal tract, breasts, spleen, pancreas, and liver [1, 2]. Furthermore, the sites of occurrence of FLH in the oral and maxillofacial regions have included the palate, tongue, salivary gland, and cheek region [4, 5]. Adkins has initially reported the clinicopathological features of lymphoid hyperplasia in the oral mucosa in 1973 [3]. In particular, it was reported that the hard palate was the most commonly affected site. However, to the best of our knowledge, only 21 cases of affected palates have been reported [6]. In a previous report of 21 case series, FLH occurred more frequently in elderly women with an average age of 62 years (range 38–79 years). The mean size of their mass lesions was 2.5 cm (range 1–5 cm) [7]. This condition usually presents as a unilateral, painless, slow-growing, and non-ulcerated mass. These features of age, gender, size, and asymptomatic findings in the present case are almost similar to the clinical characteristics of these case series reported previously. The present case may be considered as extremely unique because it arises from the posterior maxillary site. Besides the present case, another case occurring in the maxillary site has been documented [8]. The clinicopathological characteristics of 27 cases of FLH arising in the oral cavity along with our case were summarized in the Table 1 [1, 4–16]. In case 26, the size of the lesion was not specified, but it was speculated by the surrounding bone destruction that the size was larger than the present case. In the present case, there was no invasion to maxillary bone and because the boundary was clear, complete resection was possible. Both immunohistochemical profiles were similar. Although there was a difference in clinical findings, both showed strong positive for Ki67.

**Table 1:** Clinicopathological characteristics of 27 cases of FLH arising in the oral cavity

Case	References (year)	Age (years)	Sex	Site	Size (mm)	Symptoms	Image findings	Treatment	Follow-up / Status	Immunohistochemical features		
										Germinal center	Mantle zone	Parafollicular area
1	Harsany et al. 1980 [9]	60	F	Left hard palate	40 × 20	Nonpainful growth	No bony involvement	Excision/radiotherapy	144 months	NA	NA	NA
									Reduction			
2		47	M	Right hard and soft palate	NS	Nontender swelling	NS	Excision	48 months	NA	NA	NA
									Disease free			
3		72	F	Hard palate/ Buccal space	15 × 10/	Nontender mass	NS	Excision	108 months	NA	NA	NA
					25				NS			
4		70	F	Left hard palate	18	Ill-fitting denture	NS	Radiotherapy	84 months	NA	NA	NA
									Disease free			
5	Wright and Dunsworth 1983 [10]	72	F	Right hard palate	30	Swelling	No bone invasion on CT image	Excision	24 months	NA	NA	NA
									No recurrence			
6	Bradley et al. 1987 [11]	76	F	Left hard palate	NS	Asymptom	NS	Incisional biopsy	36 months	NA	NA	NA
									Disease free			
7		73	F	Left hard palate	NS	Asymptomatic mass	NS	Incisional biopsy	96 months	NA	NA	NA
									Disease free			
8		62	F	Left hard palate	35 × 20	Swelling	NS	Incisional biopsy	41 months	NA	NA	NA
									Disease free			
9		57	F	Left soft palate	30	Swelling	NS	Excision	36 months	NA	NA	NA
									No recurrence			
10		41	M	Left soft palate	NS	Swelling	NS	Excision	39 months	NA	NA	NA
									No recurrence			
11		51	F	Left hard palate	NS	Swelling	NS	Excision	24 months	NA	NA	NA
									Recurrence			
12		60	F	Bilateral palate	Large	Swelling	NS	Incisional biopsy	120 months	NA	NA	NA
									Disease free			
13	Davila and Thompson 1988 [12]	49	F	Left hard palate	30	Slightly rising	No bony involvement	Excision	84 months	NA	NA	NA
									No recurrence			
14	Napier and Newlands 1990 [13]	38	F	Junction of hard and soft palate	10	Swelling	NS	Excision	NS	P: CD45R, CD20, CDw75, CD74, HLA-DR, κ and λ light chains	P: CD45RC, CD45R, CD20, HLADR	P: CD43, CD45RC, CD45RO
										NA	N: CD43, CD45RO, CDw75, κ and λ light chains, AAT	NA
15		79	F	Junction of hard and soft palate	Several tens	Diffuse swelling	NS	Excision	NS	P: CD45R, CD20, CDw75, κ and λ light chains	P: CD45RC,CD45R, CD20, HLA-DR	P: CD43, CD45RC, CD45RO
										N: CD45RC	N: CD43. CD45RO, CDw75, κ and λ light chains, AAT	NA
16	Mopsik et al. 1992 [14]	63	M	Right hard palate	38 × 15	Swelling	No bone invasion on CT image	Excision	NS	P: κ and λ light chains	NA	NA
										NA		
17	Menasce et al. 2001 [1]	51	M	Left hard palate	20	Swelling	No bony involvement	Excision	48 months	P: κ and λ light chains	P: Bcl2	NA
									No recurrence	N: Bcl2	NA	
18		75	M	Midline hard palate	10	Swelling	NS	Excision	24 months	NA	P: Bcl2	NA
									No recurrence	N: Bcl2	NA	
19		61	F	Multifocal	Largest 20	Swelling	NS	Excision	192 months	P: κ and λ light chains	NA	NA
									No recurrence	N: Bcl2		
20	Kolokotronis et al. 2003 [15]	74	F	Right hard palate	25	Firm swelling	No bony involvement	Excision	18 months	P: CD20	P: CD20	P: CD45RO
									No recurrence	N: Bcl2	N: Bcl2, CD45RO	NA
22	Carnelio and Rodrigues 2005 [4]	36	F	Left tongue	40 × 30	Painless ulcer	NS	Excision	48 months	NA	NA	NA
									No recurrence			
21	Kojima et al. 2005 [5]	49	F	Right cheek	10 × 10	Hard mass	NS	Excision	14 months	P: CD20, CD10, IgD, IgM, CD30	P: CD20, IgD, IgM	P: CD30, CD57
									No recurrence	N: Bcl2	NA	N: CD15, EMA
23	Jham et al. 2009 [6]	55	F	Left hard palate	50	Swelling	No bony involvement	Incisional biopsy	3 months	P: CD20, CD79a,CD10, CD21, Bcl6, CD45	P: CD20, CD79a CD45	P: CD3, CD5, CD30, CD15, CD45
									Disease free			
										N: Bcl2	N: Bcl2	NA
24	Gordón-Núñez et al. 2012 [7]	70	F	Right soft palate	20	Nodular mass	No bony involvement	Excision	8 months	NA	P: Bcl2	NA
									No recurrence	N: Bcl2	NA	
25	Anjomsboaa et al. 2013 [16]	46	F	Right hard palate	20 × 15	Nontender	No intraosseous radiolucency	Intralesional steroid injection	7 months	P: CD20, CD3, CD5, CD10	P: CD20, Bcl2, Bcl6	P: CD20, Bcl2, CD3, CD5, Bcl6
						firm mass			No recurrence			
										N: Bcl2	N: CD3,CD5	NA
26	Hanemann et al. 2017 [8]	24	M	Right maxilla	NS	Swelling	Bone invasion on CT image	Curettage	36 months	P: Bcl6, CD20, CD21, CD10, CD79a, Ki67	P: CD20	P: CD3, CD5, CD20, CD138
									No recurrence			
										N: Bcl2	N: Bcl2	NA
27	Present case	51	F	Left maxilla	38 × 23 × 17	Palpation mass	No bone invasion on CT image	Excision	12 months	P: Bcl6, CD20, CD21, CD10, CD79a, Ki67	P: CD20	P: CD3, CD5, CD15, CD30
									No recurrence			
										N: Bcl2	N: Bcl2	NA

*FLH* follicular lymphoid hyperplasia, *F* female, *M* male, *NS* not stated, *P* positive, *N* negative, *NA* not applicable, *AAT* α1-antitrypsin, *EMA* epithelial membrane antigen

The specific etiology and pathogenesis of FLH have not been fully clarified to date. It has been described that this condition might represent a primary reactive lymphoid proliferation induced by some unknown antigenic stimulation or chronic irritation, for example, from a partial denture [10]. However, a source of persistent chronic irritation from a removable denture was not present in a large number of patients [1]. An association with Sjögren’s syndrome was not observed, and an association with HIV infection or any other infectious diseases has not been documented. On the other hand, the Epstein-Barr virus may be associated with an unusual form of aggressive and persistent FLH that contains clonal rearrangements of DNA [17].

Regarding the clinical differential diagnoses of the mass in the present case, salivary gland tumors, duct-associated lymphoid tissue, mesenchymal tumors, metastatic tumors, and cheek abscesses were considered initially. When occurring in the palate, some lymphoid lesions including malignant lymphoma can be easily considered in making the differential diagnoses. It is considered that the major importance of FLH is their similarity to oral lymphomas [18]. Moreover, 25% of non-Hodgkin lymphomas are extranodal, with 3–4% of all cases being located in the head and neck [19].

Considering all of the above-mentioned aspects, the definitive diagnosis of FLH is finally based on pathological examination. Histologically, FLH usually consists of multiple well-circumscribed lymphoid follicles with a clear demarcation of the germinal center and mantle zone. The majority of the lymphoid follicles have germinal centers, and some of these germinal centers are hyperplastic. The germinal centers consist of a mixture of small and large lymphoid cells, with both cleaved and uncleaved cells. Tingible body macrophages are also scattered in the germinal centers. The mantle zone contains small mature B lymphocytes and plasmacytic lymphocytes. There are variable numbers of B lymphocytes, T lymphocytes, and immunoblasts in the parafollicular area. However, Jham et al. have suggested that histological features were not always characteristic [6]. From their report, a vague nodular proliferation with indistinct germinal centers was observed. This pattern was highly suggestive of follicular lymphoma or lymphoma of the mucosa-associated lymphoid tissue (MALT). Kolokotronis et al. also described that there were some cases in which the differential diagnosis of lymphoma could be very difficult [15]. Not only indistinct germinal centers but also ill-defined mantles and a lack of tingible body macrophages were apparent. When such cases are encountered, further laboratory examination is required to assume a diagnostic process for lymphoma.

The histological diagnosis of FLH should be supported by an immunohistochemical analysis. Regarding the immunohistochemical findings of FLH, lymphoid follicles show positivity for CD20, CD21, CD10, CD79a, and Bcl6. Immunostaining for Bcl2 protein showed positivity in the mantle zone but negativity in the germinal center [1, 7]. The parafollicular areas usually revealed positivity for CD3, CD5, CD15, and CD30. In lymphoma, most neoplastic cells were reported to be positive for Bcl2 in the follicular center [20]. Another report describing 15 cases of extranodal follicular lymphoma indicated that the neoplastic follicle center cells showed coexpression of CD20 with CD10 (13/15 cases) and/or Bcl6 (15/15 cases) [21]. Bcl2 protein was detected in 9 out of the 15 cases [21]. It has also been shown that the Bcl2 oncogene is commonly activated by chromosomal translocations and that B cells undergo neoplastic transformation. This step affects the tumorigenesis of B cell malignancy [20]. Therefore, immunostaining for Bcl2 protein could be a useful marker in the differential diagnosis between FLH and lymphoma. However, it has been documented that 10–15% of follicular lymphoma was negative for Bcl2 [8]. Whereas, CD10-positive cells were also observed in the follicle center of lymphoma as well as Bcl2 [20]. CD10 is a 100-kd cell-surface metalloproteinase that appears to be involved in activation and deactivation of peptides through proteolytic cleavage [22, 23]. CD10 expresses on the surface of germinal center cells and lymphomas derived from these cells [24]. It has been suggested that the intensity of CD10 expression can be a useful maker for differentiating between follicular lymphoma and reactive follicular hyperplasia [24, 25]. However, clear expression of CD10 was observed in germinal centers in this case, so it is considered difficult to differentiate only the intensity of expression. In particular, it may be important to distinguish from CD10 expression of interfollicular neoplastic cells except germinal center. In order to arrive at definitive diagnosis, it is necessary to differentiate between FLH and neoplastic lymphoid proliferation with nodular pattern. Therefore, in addition to morphological features, it is important to evaluate immunophenotypes by immunohistochemistry.

The common treatment of FLH is surgical excision of the lesions. Radiotherapy has also been applied as treatment in a single case [9]. In the present case, the occurrence was from a very rare site, thus biopsy was initially undertaken and then a definitive diagnosis of FLH was established. Thereafter, the lesion was completely excised. A small number of patients have developed recurrences after local excision but they have not shown any evidence of malignization after long-term follow-up [1]. Although a prolonged follow-up has not shown any evidence of a malignant process, the malignant transformation of FLH into lymphoma in the skin and liver has been reported [10, 26]. Although no malignant transformation has been reported, one multisite case within the oral cavity was found to represent MALT-type lymphoma [3].

In conclusion, a definitive diagnosis of FLH can be made by careful pathological examination. Nevertheless, there are several cases that may be confused with the diagnosis of lymphoma in terms of histopathological features. In such cases, additional laboratory examinations and molecular analysis may be needed to make an accurate diagnosis. In particular, immunohistochemistry should be considered as an important part for obtaining definitive diagnosis. Although FLH usually has a good prognosis by long-term follow-up, it would be noteworthy that the possible of malignant transformation exist slightly.

## Data Availability

All relevant data are within the paper.

## Abbreviations

CT: Computed tomography
FLH: Follicular lymphoid hyperplasia
MALT: Mucosa-associated lymphoid tissue
